# Identification of learning needs of patients hospitalized at a University Hospital

**DOI:** 10.12669/pjms.306.5711

**Published:** 2014

**Authors:** Sehrinaz Polat, Selda Celik, Habibe Ayyildiz Erkan, Kamber Kasali

**Affiliations:** 1 Sehrinaz Polat, PhD, Directorate of Nursing Services, Istanbul University, Istanbul Faculty of Medicine, Istanbul, Turkey.; 2 Selda Celik, PhD, Division of Endocrinology and Metabolism, Istanbul University, Istanbul Faculty of Medicine, Istanbul, Turkey.; 3 Habibe Ayyildiz Erkan, M.Sc., Directorate of Nursing Services, Istanbul University, Istanbul Faculty of Medicine, Istanbul, Turkey.; 4 Kamber Kasali, M.Sc., Department of Biostatistics and Medical Informatics, Istanbul University, Istanbul Faculty of Medicine, Istanbul, Turkey.

**Keywords:** Hospital Discharge, Learning Needs, Hospitalized Patient, Nurse

## Abstract

**Objectives:** This study was designed as a descriptive trial aimed at identifying learning needs of patients hospitalized at a university hospital and understanding whether these learning needs vary depending on certain patient characteristics.

**Methods:** The study sample consisted of 1190 patients/caregivers hospitalized at all departments except for the psychiatry clinic, who were planned to be discharged from the hospital. Data were collected using Patient Information Form and the Patient Learning Needs Scale (PLNS).

**Results:** The evaluation of responses to the PLNS and its subscales revealed that the mean scores were 26.93±10.62 for drugs; 26.15±11.43 for activities of living; 19.78±5.54 for community and follow up; 16.86± 5.47 for feelings related to condition; 34.3±6.99 for treatment and complications; 28.20±7.40 for enhancing quality of life; and 13.64±6.54 for skin care. The PLNS total mean score was 165.95±45.44.

**Conclusion** **:** According to the evaluation of the PLNS total score, patient learning needs vary depending on age, gender, occupation, level of education, the departments at which the respondent is treated, and whether the respondent is the patient him/herself or the caregiver.

## INTRODUCTION

The pattern of health care services has been changing, with decreased duration of hospital stay and increased responsibilities of patients and family/caregivers regarding self care, which requires the provision of necessary education to patients and family/caregivers and the delivery of this education in a systematic, problem solving and qualified way. Accordingly, patient education, a specific type of health education, aims at protecting the patient from complications or other health problems associated with the disease and helping the patient to become self-sufficient in his/her physical, psychological and social life, in the shortest time possible, depending on the patient’s ability. Patient education, with its primary focus on the pathophysiology and treatment of the disease recently, has concentrated today on patients and their families taking more responsibility for their own care, protection from diseases and improvement of their health.^1^

The identification of the patient’s health care problems also enables the nurse to determine his/her learning needs. Learning needs vary depending on the stages of the process of patient compliance.^1^ Several studies, aiming at the determination of patient learning needs,^2^^-^^6^ have reported the primary learning needs to be complications and medications whereas other studies^7^^-^^9^ have reported that the most important learning needs were about activities of living and enhancing quality of life. A number of studies^3^^,^^7^^,^^10^ have reported that the least important learning needs were about skin care, feelings related to condition and community and follow up. Therefore, during the identification of patient learning needs, patients and their caregivers’ questions pertaining to health/disease should be evaluated and their level of comprehension of current health condition, effects of the disease, problems and prognosis should be determined. Necessary knowledge and skills for the patient to comprehend future practices, and to maintain his/her independence to meet his/her own self-care should be identified.^1^ The education should involve patients, their families/relatives and caregivers and cover the topic of implementation of follow up care at home.

The purpose of this study was to identify learning needs of patients hospitalized at a university hospital and to determine whether these learning needs vary depending on patients’ certain characteristics.

## METHODS

This is a descriptive study conducted methodologically. The study included 1190 patients and/or caregivers conscious and willing to participate in the study who had been hospitalized in different departments of Istanbul University Faculty of Medicine between November 2011-June 2012 and who were planned to be discharged from the hospital.

***Evaluation of the data: ***Dataset normality was tested using the Shapiro Wilk-Tests and histogram graphics. Data are presented as mean standard deviation, median, minimum-maximum, frequency and percentage. Cronbach’s alpha coefficient was used to evaluate the internal reliability of the patient learning needs scale (Cronbach’s alpha coefficient ranged between 0,724 and 0,982). Parametric tests were used for the evaluation of data. For the data analysis, NCSS (Number Cruncher Statistical System) 2007 and PASS (Power Analysis and Sample Size) 2008 statistical software (Utah, USA) were used. Besides descriptive statistical methods, one-way Anova test was used to compare normally distributed variables between independent three or more groups and Tukey’s HSD test was used for Post Hoc analysis whereas the comparison of independent two groups was performed using the student t test. The Pearson correlation analysis was conducted to examine the relationships among the variables.

***Data collection tools: ***The data collection tools used in this study included the Patient Information Form and The Patient Learning Needs Scale (PLNS). The patient information form includes questions related to age, gender, level of education, occupation and duration of hospitalization of the participating patient and/or his/her caregivers, the departments at which the participant is treated and questions asking whether they would recommend the hospital to others. The PLNS was first developed by Bubela et al. in 1990 to identify the importance of information in patients.^11^ In this study, the Turkish version of the Patient Learning Needs Scale, whose validity and reliability testing was conducted by Çatal and Dicle,^12^ was used after the necessary permission was obtained. The scale consists of 7 subscales (medications, activities of living, community and follow up, feelings related to condition, treatment and complications, enhancing quality of life, skin care) and a total of 50 items. Each item is scored on a Likert type scale of 1 (not important) to 5 (extremely important). The total score ranges between 50 and 250.

Cronbach’s alpha for the PLNS was reported by Bubela et al. to be 0.95 for the total scale and to range between 0.69 and 0.88 for the subscales.^11^ In their study, Catal and Dicle reported Cronbach’s alpha values to be 0.93 for the total scale and to range between 0.57 and 0.83 whereas Cronbach’s alpha values were found to be 0.96 for the total scale and to range between 0.76 and 0.95 for the subscales in our study.^12^

***Research Ethics: ***Prior to the study, the approval of the General Directorate and the Ethics Committee of Istanbul University Faculty of Medicine was obtained. The participants were asked to complete the questionnaire form in 24-48 hours before hospital discharge. The study was conducted in accordance with Helsinki Declaration.

## RESULTS

Of the patients and their caregivers constituting the study sample, 66.2% were treated in the Surgical departments and 33.8% in the departments of Internal Medicine. Of all participants, 55.6% were female, 26.2% were aged 61 years or older, 40.8% were housewives and 48.9% had primary education. Of all participants, 58.7% were hospitalized for between 0-10 days (Table-I).

The total mean score of all questions answered by the participants was 165.95±45.44, with the maximum score being 34.38±6.99 for the treatment and complications subscale and the minimum score being 13.64±6.54 for the skin care subscale (Table-II).

According to the age groups, there was a statistically significantly high difference in medications, activities of living; community and follow up, skin care and PLNS total scores among the participants. On the other hand, no statistically significant differences were noted for participants’ scores for the PLNS subscales of ‘feelings related to condition’, ‘treatment and complications’ and ‘enhancing quality of life”. According to paired comparisons; participants aged between 31 and 40 years had statistically significantly higher scores for the medications subscale compared to those in the ≤30-year, 51-60 year and ≥61-year age groups and had statistically significantly higher scores for the activities of living scale in comparison to those in the ≤30-year, 51-60 year and ≥61-year age groups. The participants included in the 41-50 year age group had statistically significantly higher scores for activities of living in comparison to those in the ≥61-year age group. The participants in the 31-40-year and 41-50 year age groups had statistically significantly higher scores for the community and follow up subscale in comparison to those in the ≥61-year age group. The PLNS total score was found to be significantly higher than the ≥61 year age group. The 31-40 year age group had significantly higher scores than the 51-60-year age group. The scores for the skin care subscale were significantly higher in the ≤30 year, 31-40-year and 41-50 year age groups in comparison to those in the ≥61 year age group. The participants in the 31-40 year age group had significantly higher scores than those in the 51-60 year age group (Table-III).

The difference among the scores of the participants for the PLNS subscale of “medications” was not statistically significant according to gender, but was close to the margin of statistical significance. It is noteworthy that female participants’ had higher scores for the medications subscale than males. Female participants had significantly higher scores for the subscales of activities of living, community and follow up, feelings related to condition, enhancing quality of life and skin care than males. The difference among the participants’ scores for the PLNS subscale of “treatment and complications” according to gender was not statistically significant but was close to the margin of statistical significance. It is noteworthy that female participants had higher scores for the “medications” subscale than males. A statistically significantly high difference was found among the PLNS total sores of participants according to gender. Females had significantly higher total scores than males.

With regard to occupations, there were statistically significant differences between the participants’ total scale scores and mean scores for the subscales of medications, activities of living, community and follow up, treatment and complications, enhancing quality of life, and skin care. The participants in the “self-employed” group had statistically significantly higher scores for the “medications” than housewives, retired participants and workers and had significantly higher scores for the “activities of living” subscale than housewives, retired participants and workers. In addition, they had statistically significantly higher scores for the “community and follow up” subscale than retired participants, for the “enhancing quality of life” subscale than workers, for the “skin care” subscale than retired participants and had significantly higher total scores than retired participants and workers. Moreover, the participants in the “self-employed” group had significantly higher total scores than retired participants and workers.

With regard to the level of education, there were statistically significant differences among the participants’ scores for the subscales of medications, activities of living, community and follow up, treatment and complications, enhancing quality of life, and skin care and PLNS total mean scores. Participants with a university degree had statistically significantly higher scores for the “medications” subscale than participants with a primary education, for the “activities of living” subscale than those with a primary education, for the “community and follow up” subscale than those with a primary education, for the “treatment and complications” subscales than participants with a primary or high school education, for the “enhancing quality of life” subscale than those with a primary or high school education, for the “skin care” subscale than those with a primary education and had higher PLNS total scores than those with a primary education. PLNS total and subscale scores did not reach statistical significance in terms of the duration of hospitalization.

Participants’ scores for the PLNS subscales of medications, activities of living and their total scores were significantly higher in the departments of internal medicine whereas their scores for the subscale of feelings related to condition were significantly higher in the surgical clinic. The scores for the subscales of community and follow up, treatment and complications, enhancing quality of life and skin care did not reach statistical significance in terms of clinics.

The scores for the PLNS subscales of medications, activities of living, skin care and the total scores showed statistical significance in terms of whether the respondent is the patient him/herself or the caregiver. The PLNS scores of the caregivers for the subscales of medications, activities of living, and skin care were significantly higher than those of the patient him/herself and those of patient/caregiver together. There was no statistical significance between the PLNS scores of the patients only and those of patients/caregivers together. Caregivers had significantly higher scores for the subscales of community and follow up and enhancing quality of life than patients only. The scores for the feelings related to condition subscale did not reach statistical significance according to the participants responding to the questionnaire.

## DISCUSSION

It is evident that studies on the identification of patients’ learning needs have reported different results regarding patients’ priority of learning needs. In studies addressing learning needs of patients and caregivers, the most important learning needs were about treatment and complications, medications and activities of living.^2^^-^^18^ However, some studies have reported different results.^9^^,^^17^ In this study, the patients had the highest learning needs in the domains of treatment and complications, enhancing quality of life, medications, activities of living, community and follow-up, feelings related to condition and skin care, respectively.

We also examined whether PLNS subscale and total mean scores varied depending on gender. It was discovered that females had higher scores for the PLNS subscales of activities of living, community and follow up, feelings related to condition, enhancing quality of life and skin care and had higher PLNS total mean scores than males. Previous studies have reported that females expressed higher learning needs than males.^2^^,^^4^^,^^8^^,^^9^^,^^14^^,^^18^

**Table-I:** Descriptive characteristics of the participants

**Variables**		**n**	**%**	**Variables**		**n**	**%**
Survey respondents age	≤ 30 age	285	23.9	**Profession**	Housewives	486	40.8
	31-40 age	174	14.6		Retired	310	26.1
	41-50 age	197	16.6		Student	73	6.1
	51-60 age	222	18.7		Officer	61	5.1
	≥ 61 age	312	26.2		Worker	125	10.5
					Other	135	11.3
Age of Patients	≤ 18 age	123	10.3				
	19-30 age	162	13.6	**Department**	Internal medicine	402	33.8
	31-40 age	174	14.6		Surgical clinic	788	66.2
	41-50 age	197	16.6				
	51-60 age	222	18.7				
	≥ 61 age	312	26.2				
Gender				**Duration of Hospitalition**	0-10 day	699	58,7
	Female	674	56.6		11-20 day	274	23.0
	Man	516	43.4		21-30 day	111	9.3
					> 30 day	106	8.9
Education	Illiterate	78	6.6				
	Primary education	582	48.9				
	High school	345	29.0				
	University	185	15.5				

**Table-II:** Distribution of the patient learning needs scale scores

	**Patient education requirements**		
	**Mean±SD**	**Median**	**Min-Max**
Drugs	26.93±10.62	29.0	8-40
Living activity	26.15±11.43	27.0	9-45
Society and monitoring	19.78±5.54	19.0	6-30
Condition related emotions	16.86±5.47	18.0	5-25
Treatment and complications	34.38±6.99	34.0	9-45
Quality of life	28.20±7.40	28.0	8-40
Skincare	13.64±6.54	12.0	8-40
Total	165.95±45.44	155.0	50-250

**Table-III:** The evaluation of learning needs of the participants according to demographic characteristics

	**Medications**	**Activities of living**	**Community and follow**	**Feelings related to condition**	**Treatment and Complications**	**Quality of life**	**Skin care**	**Total**
Age (year) ap	*0.001* **	*0.001* **	*0.008* **	*0.223*	*0.07*	*0.431*	*0.001* **	*0.001* **
Gender bp	*0.057*	*0.001* **	*0.007* **	*0.007* **	*0.059*	*0.018* *	*0.001* **	*0.001* **
Occupation ap	*0.006* **	*0.001* **	*0.018* *	*0.687*	*0.013* *	*0.038* *	*0.002* **	*0.003* **
Education ap	*0.003* **	*0.004* **	*0.038* *	*0.152*	*0.007* **	*0.020* *	*0.012* *	*0.002* **
Duration of hospitalization ap	*0.23*	*0.058*	*0.928*	*0.528*	*0.836*	*0.634*	*0.901*	*0.858*
Department bp	*0.001* **	*0.001* **	*0.794*	*0.001* **	*0.626*	*0.059*	*0.825*	*0.001* **
Participating in Research (Patient/ Cargivers ap)	*0.001* **	*0.001* **	*0.020* *	*0.485*	*0.001* **	*0.048* *	*0.001* **	*0.001* **

a Oneway Anova Test

b Student t Test

** p<0.01

* p<0.05

In addition to several studies in the literature reporting differences in patients’ learning needs in relation to the age group,^2^^,^^8^^,^^12^^,^^17^^,^^18^ no statistically significant differences were found among PLNS total and subscale mean scores according to patients’ age groups.^7^^,^^9^^,^^14^^,^^15^ Frederics et al. reported that perceived learning needs for patients younger than 45 years were higher than older patients; ^17^ Ilk reported that learning needs for treatment and complications were lower in patients aged 50 years or younger than in those aged between 51 and 60 years and those aged 61 years or older;^8^ Johansson et al. reported that learning needs were higher in patients aged between 60 and 69 years than in those younger than 40 years;^2^ Smith and Lines reported that patients aged 65 years or older had found information about “the support and care in the community” more important than those aged 64 years or younger.^13^ In this study, statistically significant differences were found between learning needs according to age. In this study, statistically significant differences were noted in PLNS scores and PLNS subscales of medications, activities of living, community and follow up and skin care according to age. In general, learning needs were found to be lower in those older than 61 years of age compared to younger ones. 

Previous studies have reported differences in patients’ learning needs in relation to the level of education.^2^^,^^9^^,^^14^^,^^18^ Similar to our findings, Tan et al. reported that patients with a high school or higher level of education had higher mean scores for medications and enhancing quality of life compared to those in other educational level groups,^18^ which may be associated with increased awareness and expectations in individuals with higher level of education.

Despite the study results suggesting that learning needs vary depending on occupations,^9^^,^^13^^,^^15^ some studies have reported no difference based on occupations, as in study by Ozel.^14^ Demirkiran and Uzun reported that PLNS mean total scores were higher in housewives but lower in officers compared to those in other occupational groups and that the mean scores for activities of living, community and follow up, treatment and complications and skin care were higher in housewives than those in others.^9^ Smith and Lines reported that retired patients desired more information concerning community and follow up than their employed counterparts.^13^

In a study by Tan et al., individuals with no hospital experience had higher mean scores for the treatment and complications subscale than those with hospital experience.^18^ A study by Ozel reported that patients with a duration of hospitalization of longer than 16 days had high scores for the subscales of medications and feelings related to condition and that the difference reached statistical significance. In this study, our results showed that PLNS total and subscale scores did not reach the level of statistical significance according to the duration of hospitalization. In the studies by Jacops and Demirkiran and Uzun, no statistically significant differences were noted in duration of hospitalization between PLNS total and subscale scores, which is consistent with our findings.^7^^,^^9^

## CONCLUSION

The evaluation of the PLNS total score revealed that patient learning needs vary depending on age, gender, occupation, level of education, the departments at which the respondent is treated, and whether the respondent is the patient him/herself or the caregiver. For the identification of learning needs of individuals with lower level of education, questions can be addressed verbally and visual materials that include images can be of assistance. Learning needs of not only the patient but also of present and future caregivers should also be identified. The discharge process should be individualized for each patient and be planned according to each patient’s individual characteristics. Nurses should be given in-service training on the discharge planning process and be encouraged to become fully aware of their educational roles. 

## References

[1] Tasocak G (2012). Patient education.

[2] Johansson K, Hupli M, Salantera S (2002). Patients’ learning needs after hip arthroplasty. J Clin Nurs.

[3] Tasdemir N, Guloglu S, Turan Y, Cataltepe T, Ozbayir T (2010). Learning needs of neurosurgery patient. J Neurolog Sci.

[4] Uzun O, Ucuzal M, Inan G (2011). Post-discharge learning needs of general surgery patients. Pak J Med Sci.

[5] Potter P, Deshields T, Kuhrik M, Kuhrik N, O’Neill J, Rihanek A (2010). An analysis of educational and learning needs of cancer patients and unrelated family caregivers. J Canc Educ.

[6] Yu M, Chair SY, Chan C WH, Li Xiaomei, Choi KC (2012). Perceived learning needs of patient with heart failure in china: a cross-sectional questionnaire survey. Contemporary Nurse.

[7] Jacobs V (2000). Informational needs of surgical patients following discharge. Applied Nurs Res.

[8] Ilk A (2010). Determining the learning needs of the patients who have chronic disease and stay in the internal diseases clinic [Master Thesis].

[9] Demirkiran G, Uzun O (2012). Post-discharge learning needs of patients who had undergone coronary artery bypass grafting surgery. J Ege Uni Nurs Faculty.

[10] Eshah NF (2011). Jordanian acute coronary syndrome patients’ learning needs: implications for cardiac rehabilitation and secondary prevention program. Nurs Health Sci.

[11] Bubela N, Galloway S, McCay E, McKibbon Ann, Nagle L, Pringle D (1990). The patient learning needs scale: reliability and validity. J Adv Nurs.

[12] Catal E, Dicle A (2008). A validity and reliability study of the patient learning needs scale in Turkey. Dokuz Eylul Uni School Nurs Electronical J.

[13] Smith J, Liles C (2007). Information needs before hospital discharge of myocardial infarction patient: A comparative, descriptive study. J Clin Nurs.

[14] Ozel S (2010). Determination of information needs after the discharge of the patients who had surgica [Master].

[15] Erdogan M (2012). Determining the educational requirements of the patients with open-heart surgery [Master].

[16] Orgun F, Sen G (2012). Determination of the learning needs of hospitalized patients in surgical units of state hospital. J Sports Med Sci.

[17] Fredericks S, Guruge S, Sidani S, Wan (2012). Patient demographics and learning needs: examination of relationship. Clin Nurs Res.

[18] Tan M, Ozdelikara A, Polat H (2013). Determination of patient learning needs. FN J Nurs.

